# Biological and Functional Changes in Healthy Adult Smokers Who Are Continuously Abstinent From Smoking for One Year: Protocol for a Prospective, Observational, Multicenter Cohort Study

**DOI:** 10.2196/12138

**Published:** 2019-06-07

**Authors:** Cam Tuan Tran, Loyse Felber Medlin, Nicola Lama, Brindusa Taranu, Weeteck Ng, Christelle Haziza, Patrick Picavet, Gizelle Baker, Frank Lüdicke

**Affiliations:** 1 Philip Morris International Science and Innovation Philip Morris Products SA Neuchâtel Switzerland

**Keywords:** smoking cessation, smoking, tobacco, harm reduction, tobacco products, biomarkers, metabolic networks, pathways

## Abstract

**Background:**

The harm of smoking results mainly from long-term exposure to harmful and potentially harmful constituents (HPHCs) generated by tobacco combustion. Smoking cessation (SC) engenders favorable changes of clinical signs, pathomechanisms, and metabolic processes that together could reduce the harm of smoking-related diseases to a relative risk level approximating that of never-smokers over time. In most SC studies, the main focus is on the quitting rate of the SC program being tested. As there is limited information in the literature on short to multiple long-term functional or biological changes following SC, more data on short to mid-term favorable impacts of SC are needed.

**Objective:**

The overall aim of the study was to assess the reversibility of the harm related to smoking over 1 year of continuous smoking abstinence (SA). This has been verified by assessing a set of biomarkers of exposure to HPHCs and a set of biomarkers of effect indicative of multiple pathophysiological pathways underlying the development of smoking-related diseases.

**Methods:**

This multiregional (United States, Japan, and Europe), multicenter (42 sites) cohort study consisting of a 1-year SA period in an ambulatory setting was conducted from May 2015 to May 2017. A total of 1184 male and female adult healthy smokers, willing to quit smoking, were enrolled in the study. Nicotine replacement therapy (NRT) was provided for up to 3 months upon the subject’s request. SC counseling and behavioral support were continuously provided. Biomarkers of exposure to HPHCs and biomarkers of effect were assessed in urine and blood at baseline, Month 3, Month 6, and Month 12. Cardiovascular biomarkers of effect included parameters reflecting inflammation (white blood cell), lipid metabolism (high-density lipoprotein cholesterol), endothelial function (soluble intercellular adhesion molecule-1), platelet function (11-dehydrothromboxane B2), oxidative stress (8-epi-prostaglandin F2 alpha), and carbon monoxide exposure (carboxyhemoglobin). Respiratory biomarkers of effect included lung function parameters and cough symptoms. The biomarkers of effect to evaluate genotoxicity (total 4-(methylnitrosamino)-1-(3-pyridyl)-1-butanol) and xenobiotic metabolism (cytochrome P450 2A6 activity) were also assessed. Continuous SA was verified at each visit following the actual quit date using self-reporting and chemical verification. Safety assessments included adverse events and serious adverse events, body weight, vital signs, spirometry, electrocardiogram, clinical chemistry, hematology and urine analysis safety panel, physical examination, and concomitant medications.

**Results:**

In total, 1184 subjects (50.1% male) were enrolled; 30% of them quit smoking successfully for 1 year. Data analyses of the study results are ongoing and will be published after study completion.

**Conclusions:**

This study provides insights into biological and functional changes and health effects, after continuous SA over 1 year. Study results will be instrumental in assessing novel alternative products to cigarettes considered for tobacco harm reduction strategies.

**Trial Registration:**

ClinicalTrials.gov NCT02432729; http://clinicaltrials.gov/ct2/show/NCT02432729 (Archived by WebCite at http://www.webcitation.org/78QxovZrr)

**International Registered Report Identifier (IRRID):**

DERR1-10.2196/12138

## Introduction

Cigarette smoking is the leading cause of preventable deaths worldwide and is associated with increased risk of pulmonary disease, cardiovascular disease (CVD), and other serious diseases, such as cancer [1]. The World Health Organization (WHO) has estimated that there will be 1.5 billion smokers globally by 2050 [2]. Preventing smoking initiation and increasing smoking cessation (SC) can reduce the number of deaths and is a public health priority. However, complete and permanent SC is challenging for many current smokers. Most smokers (55%) do not try to quit, and those who do try often relapse to smoking. Only about 5% are able to quit smoking for 1 year or longer [1].

It is widely recognized that the harm associated with smoking results mainly from long-term exposure to the harmful and potentially harmful constituents (HPHCs) contained in cigarette smoke, generated by combustion of tobacco and not from nicotine itself [3], as stated by the Royal College of Physicians “most of the harm caused by smoking arises not from nicotine but from other components of tobacco smoke” [4]. Exposure to HPHCs leads to molecular changes causing perturbations in biological mechanisms, which in turn cause cell and tissue damage, physiological changes, and disease manifestation to the individual, ultimately leading to population burden. Smoking affects multiple organ systems, disease pathways, and mechanisms, such as inflammation, oxidative stress, platelet activation, and lipid metabolism, simultaneously [5].

Owing to the abundant concurrent processes in disease pathways, there is no single biomarker that is considered as a validated surrogate measure reflecting the biological processes, physiological system, and/or a mechanism of action that is associated with, or actually known to contribute to, smoking-related diseases. Numerous epidemiological studies have shown that most of the smokers who quit smoking benefit from a gradual and significant reduction of harm and risk of smoking-related diseases over time, as SC favorably reverses many of the adverse functional and biological changes associated with smoking [6-8]. It has been demonstrated that long-term smoking abstinence (SA) results in reduced blood levels of hemostatic and inflammatory markers, such as white blood cell (WBC) or fibrinogen, which are important determinants in the subsequent development of cardiovascular or chronic obstructive pulmonary disease, reverting to levels of never-smokers [9]. Furthermore, SC curtails the decline in forced expiratory volume in 1 second (FEV_1_) predicted and improves respiratory function [5,10]. Additional health benefits have also been described, including favorable changes in oxidative stress (eg, 8-epi-prostaglandin F2 alpha [8-epi-PGF_2__α_]) [11], lipid metabolism (high-density lipoprotein [HDL] cholesterol) [12], endothelial function (eg, soluble intercellular adhesion molecule-1 [sICAM-1]) [13], and platelet function (eg, 11-dehydrothromboxane B2 [11-dehydro-TXB2]) [14].

Despite the substantial amount of SC studies in the literature, the main focus of these studies has been the successful quitting rates as a result of SC treatment rather than on evaluating short- and long-term (up to 1 year and beyond) functional and biological changes in the body upon continuous SA. Given the need for additional data to bridge this evidence gap, providing broader and deeper insights into the clinical benefits upon SC, we conducted a study in adult healthy smokers who were continuously abstinent from smoking for 1 year. The overall aim of our study was to assess the reversibility of smoking-related harm after continuous SA.

Using available epidemiological data reporting quantitative estimates of the association with CVD, respiratory diseases and cancer in smokers and reversibility upon SC within a 1-year time frame, a set of biological and functional parameters, identified as biomarkers of effect and biomarkers of exposure to HPHCs, were selected based on predefined criteria and assessed in this study. Covering multiple pathways involved in the pathogenesis of smoking-related diseases, the selected parameters will provide an overall understanding of how SC triggers favorable changes and the time frame of reversibility from mechanistic pathways that are commonly involved in the onset and progression of smoking-related diseases.

Offering smokers nicotine delivery products, with the potential to reduce the risk of smoking-related diseases, as a replacement for cigarettes is an emerging approach for smoking harm reduction strategy [15].

To assess reduced risk potential, the Institute of Medicine of the National Academies recommends the use of appropriately designed studies to establish whether the use of novel alternatives to cigarettes, such as heat-not-burn tobacco products or nicotine-containing e-vapor products, reduces exposure to toxicants or induces positive changes in surrogate markers [16]. If the magnitude and time frame of positive changes in surrogate markers approximate those observed of SC, then that would provide pivotal scientific evidence to demonstrate the reduced risk potential of the alternative products. This study will provide a comprehensive assessment of the favorable health effects of SC on multiple short- and long-term biological and functional endpoints evaluated over 1 year and could serve as a benchmark to assess novel alternatives to cigarettes considered for tobacco harm reduction strategies for smokers who would otherwise continue to smoke.

## Methods

### Study Design

**Figure 1:**
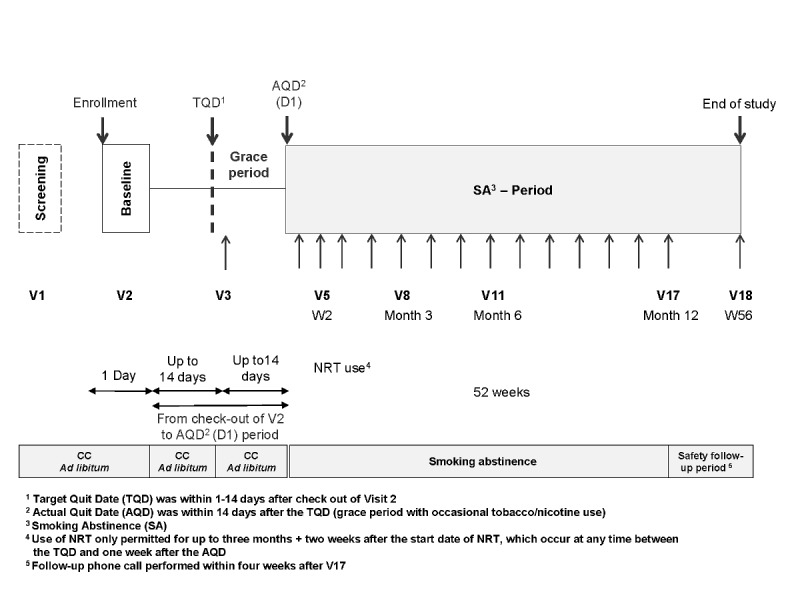
Study design and timeline. Target quit date (TQD) was within 1-14 days after check out of Visit 2; actual quit date (AQD) was within 14 days after the TQD (grace period with occasional tobacco/nicotine use). Nicotine replacement therapy (NRT) was only permitted for up to three months + two weeks after the start date of NRT, which occurred at any time between the TQD and one week after the AQD. CC: cigarettes; SA: smoking abstinence; V: visit; W: week.

This 56-week, multiregional, multicenter, ambulatory study was conducted at 42 sites in the United States, Japan, and Europe, and it is registered at ClinicalTrials.gov (identifier NCT02432729). The institutional review boards or independent ethics committees for each participating institution granted ethical approval. The study followed the principles defined by the International Conference on Harmonization Good Clinical Practice, the Declaration of Helsinki, and other applicable regulations [17,18]. No smoking control arm was included, as the endpoints were analyzed before and after SC and numerous studies have already documented such aspects in smokers [19-21]. The first subject was enrolled in the study on May 5, 2015, and the last subject completed the study on May 30, 2017. The study design is illustrated in Figure 1. Samples were collected during the baseline visit for baseline biomarker analysis. During this visit, participants were asked to define their target quit date, the date from which they would stop smoking. The target quit date had to be within 14 days after the baseline visit. The next visit, scheduled 24 to 48 hours after the target quit date, was used to determine whether the subject had stopped smoking and to provide SC support. A grace period of up to 14 days was allowed after the target quit date, during which occasional use of nicotine and/or tobacco-containing products was tolerated. Starting from the actual quit date, participants had to abstain completely from smoking and from using any nicotine or tobacco-based products other than nicotine replacement therapy (NRT; allowed for up to 3 months and 2 weeks). The period from baseline visit to the actual quit date has been established to identify participants with a higher likelihood of successful SA for 1 year.

Participants recorded their own actual quit date and provided the information to their study clinic. Visits were scheduled on a monthly basis for the duration of the 52-week study (1 year), with biomarker sample collections scheduled for the Month 3, Month 6, and Month 12 visits. The study ended after a 28-day safety follow-up period. SC support, including counseling and behavioral support, was provided throughout the study at scheduled visits and between visits as requested by the subject. Participants were allowed to use NRT to support SC if requested by the subjects. NRT was started any time between the target quit date and 1 week after the actual quit date and was permitted for up to 3 months and 2 weeks. Adverse events were collected at each visit. Participants who smoked after the actual quit date were discontinued from the study.

### Participants

All participants provided written informed consent. The study enrolled healthy adult smokers who were motivated to quit smoking within the next 30 days. Their motivation was assessed using a questionnaire based on the Prochaska stages of change [22]. The main inclusion and exclusion criteria are summarized in Textbox 1. Participants had at least 10 years of smoking history and had smoked at least 10 cigarettes per day over the last 12 months. Participants with FEV_1_ and forced vital capacity (FVC) <0.7 and FEV_1_ <80% predicted value at postbronchodilator spirometry and those with FEV_1_ and FVC <0.75 (postbronchodilator) and reversibility in FEV_1_ (both >12% and >200 mL from pre- to postbronchodilator values) were excluded from the study. There were no limitations on race or ethnicity. Stratified sampling was used to ensure adequate representation of genders (at least 40% of each sex at enrollment).

A total of 1035 participants who successfully abstained from smoking for at least 2 weeks after the actual quit date were remaining in the study, meaning the study was completed with at least 190 successful quitters.

**Inclusion and exclusion criteria of participants.**

Inclusion criteria

- Informed consent form(s) signed
- Age 30 to 65 years (inclusive)
- Positive urine cotinine test at both screening and Visit 2 (cut-off ≥200 ng/mL)
- Smoking history of at least 10 years before screening
- Smoking history of at least 10 cigarettes/day on average in the 12 months preceding screening (as reported by the subject)
- Willingness to quit smoking within the next 30 days

Exclusion criteria

- Clinically relevant gastrointestinal, renal, hepatic, neurological, hematological, endocrine, oncological, urological, pulmonary, immunological, psychiatric, or cardiovascular disorders or any other conditions that would jeopardize the safety of the participant or affect the validity of the study results
- Abnormal findings on physical examination, in the medical history, or in clinical laboratory results deemed clinically relevant by investigators (as per the common terminology criteria for adverse events)
- Acute illness (eg, upper-respiratory tract infection and viral infection) requiring treatment within 42 days before enrollment in the study
- Use of any prohibited, prescribed, or over-the-counter systemic medications within 42 days of enrollment (except for vitamins, hormonal contraceptives, and hormone replacement therapy)
- Forced expiratory volume in one second (FEV_1_)/forced vital capacity (FVC) <0.7 and FEV_1_ <80% predicted value at postbronchodilator spirometry
- FEV_1_/FVC <0.75 (postbronchodilator) and reversibility in FEV_1_ >12% and >200 mL from pre- to postbronchodilator values
- Pregnancy or breastfeeding

### Study Objectives and Endpoints

The main objective of the study was to describe the biological and functional changes in smokers who are continuously abstinent from smoking. The biomarkers of effect, including those associated with CVD, respiratory diseases, xenobiotic metabolism, and genotoxicity, are provided in Table 1. This broad range of biomarkers of effect were selected based on the predefined criteria according to the epidemiological evidence that the biomarkers of effect were associated with smoking-related diseases, sensitive to smoking status, and reversible upon SC over a period of time that was compatible with the study duration.

The biomarkers of exposure to HPHCs in smokers who continuously abstained from smoking (Table 2) were derived from published guidelines from the WHO and the US Food and Drug Administration [23,24] and according to predefined criteria, as reported previously [25].

The rate of continuous SA was determined at each visit following the actual quit date.

Safety was established by monitoring adverse events, body weight, vital signs, spirometry, electrocardiogram, hematology and clinical chemistry marker panels, urine analysis, physical examination, and concomitant medications. Adverse events were coded according to MedDRA terminology.

**Table 1:** Biomarkers of effect.

Variable, effect category		Effect
**Associated with cardiovascular disease**		
	Lipid metabolism	High-density lipoprotein cholesterol Low-density lipoprotein cholesterol Apolipoprotein A1 (Apo A1) Apolipoprotein B (Apo B) Apo B/Apo A1
	Inflammation	White blood cell count High-sensitivity C-reactive protein Homocysteine
	Platelet function	Platelet cell count Fibrinogen 11-dehydrothromboxane B2 (urine)
	Oxidative stress	8-epi-prostaglandin F2 alpha (urine) Myeloperoxidase
	Endothelial dysfunction	Soluble intercellular adhesion molecule-1 Albumin (urine)
	Acute cardiovascular effect	Carboxyhemoglobin
	Metabolic syndrome	Glycosylated hemoglobin
**Associated with respiratory diseases**			
	Spirometry	Forced expiratory volume in one second (FEV_1_) Forced vital capacity (FVC) FEV_1_/FVC Forced expiratory flow at 25–75% of the pulmonary volume (FEF25-75)
	Lung volume	Vital capacity Total lung capacity Functional residual capacity Inspiratory capacity Residual volume
	Cough	Cough symptoms (intensity and frequency) Sputum production and bothersome cough symptoms reported in cough questionnaire
	Lung sounds analysis	Computerized multichannel Stethographics and Stethos
	Gas transfer	Carbon monoxide lung diffusion capacity and rate constant
**Associated with xenobiotic metabolism**		
	—^a^	Cytochrome P450 2A6 activity
**Associated with genotoxicity**		
	—	Total 4-(methylnitrosamino)-1-(3-pyridyl)-1-butanol (Total NNAL; urine)

^a^Not applicable.

**Table 2:** Biomarkers of exposure endpoints.

Harmful and potentially harmful constituents	Biomarkers of exposure
Carbon monoxide	Carbon monoxide in exhaled breath
Nicotine	Cotinine and nicotine in plasma and nicotine equivalents in urine
1,3-butadiene	Monohydroxybutenylmercapturic acid
Acrolein	3-hydroxypropylmercapturic acid
Acrylonitrile	2-cyanoethylmercapturic acid
Benzo(a)pyrene	3-hydroxybenzo(a)pyrene
Pyrene	Total 1-hydroxypyrene
Crotonaldehyde	3-hydroxy-1-methylpropylmercapturic acid
N-nitrosonornicotine	N-nitrosonornicotine
4-aminobiphenyl	4-aminobiphenyl
Benzene	S-phenylmercapturic acid
1-aminonaphthalene	1-aminonaphthalene
2-aminonaphthalene	2-aminonaphthalene
o-toluidine	o-toluidine
Ethylene oxide	2-hydroxyethylmercapturic acid
Toluene	S-benzylmercapturic acid

### Study Measurements

Full lung function assessments, blood and urine samples for biomarkers of effect, and biomarkers of exposure analyses were conducted at baseline (Visit 2) and at 3 time points during the study (Month 3, Month 6, and Month 12 visits). Cough assessment by visual analog scale and Likert scales (intensity of cough, frequency of cough, and amount of sputum collection) were conducted at baseline (Visit 2) and at 3 time points during the study (Month 3, Month 6, and Month 12 visits). Noncompliance with continuous SA was verified as follows:

- At each visit, participants were asked to confirm their continued abstinence from smoking from the actual quit date onward (ie*,* free from tobacco product use [eg, cigarettes, pipes, cigars, and snus] or any nicotine-containing products [including electronic cigarettes] other than NRT) or continued NRT use after the allowed time frame (ie, 3 months and 2 weeks after the NRT start date).
- CO breath test (>10 pm) from Visit 4 onward.
- Urine cotinine test at each visit from Visit 10 onward (cotinine test ≥100 ng/ml).
- Free cotinine concentration (part of nicotine equivalents) in 24-h urine collected at Visit 11 (free cotinine ≥50 ng/mL).

Socioeconomic status, lifestyle, stage of change [22], and nicotine dependence [26] were assessed as baseline characteristics. One central laboratory was used for sample management and several laboratories were used for the blood and urine analyses (Covance Central Laboratory Services Inc; Celerion, Lincoln, United States; and Celerion Switzerland AG, Switzerland) using validated and fit-for-purpose methods.

### Statistical Considerations

On the basis of the results of the Lung Health Study and on the 1-year abstinence rates from English smoking treatment services [27], at least 950 smokers were required to have approximately 190 participants who successfully abstain from smoking for the duration of the study [28]. Sample size was driven by FEV_1_, which is expected to have the lowest effect size. Approximately 190 participants were needed to estimate the mean increase from baseline of 1.98 (% predicted) in FEV_1_ at Month 12, with a 90% probability of obtaining a margin of error (95% CI) of at most ±1 (% predicted). Enrolled participants who failed to abstain for at least 2 weeks were discontinued from the study.

Changes from baseline were summarized for the main analysis population, defined by quitters with no major protocol deviations impacting subject availability. Data collected after evidence of noncompliance with continuous abstinence were not included in the analysis. For analysis purposes, the concentrations of free cotinine (≥50 ng/mL) [29] and total NNAL (≥75.9 pg/mL) [30] in 24-hour urine collected at Visit 11 were included in the list of tools for continuous SA verification. This study had no formal prespecified hypotheses to be tested for statistical significance. However, a 95% CI accompanied all effect estimates.

## Results

**Figure 2:**
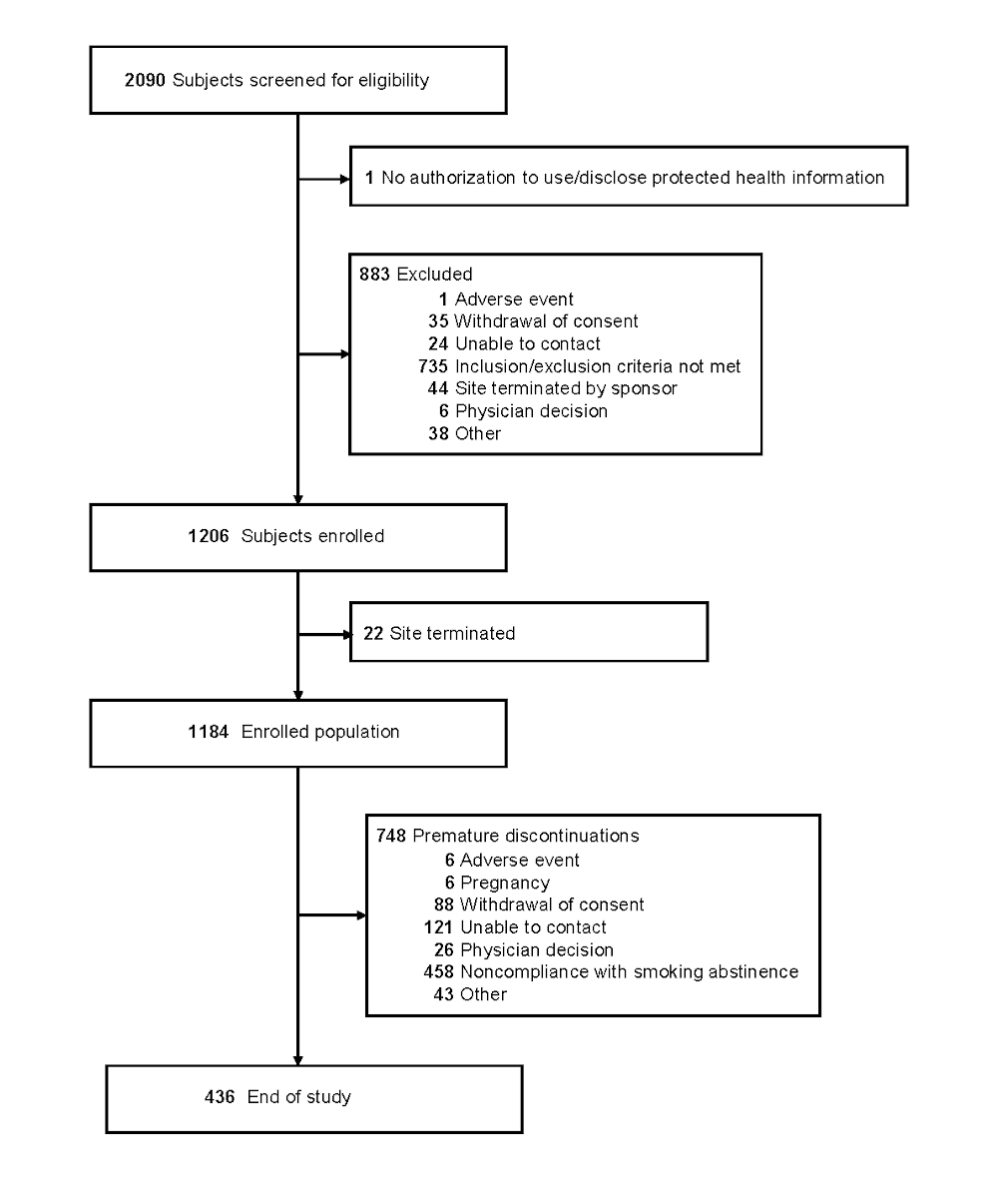
Flow chart of study participants.

A total of 2090 subjects were screened for the study (Figure 2); 1206 subjects were included in the Full Safety Population, 1184 subjects were enrolled, and of these, 358 subjects were abstinent from smoking for 1 year. The mean age of the enrolled subjects was 43.8 years, and 50.1% were male.

The study was completed in May 2017. The results of this study are under evaluation and will be published upon completion of analysis.

## Discussion

### Overview

The approach of our study is unlike other SC studies whose primary objective was to test the efficacy of an SC treatment (ie, drug or behavioral cessation support) [31-33]. This dataset will supplement the existing literature data on the effects of SC while providing prospective and comprehensive perspective on favorable health effects that occur following SC. Although several papers report favorable changes in biomarkers of effect with SC [34,35], there are very little data on 1-year observations and none with such an extensive set of endpoints for clinical risk and exposure. Changes in inflammatory markers with SC have been evaluated in previous studies. SC is reported to reduce oxidant stress and inflammation. This is evidenced by an improved urinary F_2_ isoprostane:creatinine [F_2_:Cr] ratio and decreases in WBC counts [36]. Time-dependent changes in LDL levels have been reported with cessation. LDL levels were measured in 50 smokers before cessation and at 3 months and 1 year after cessation. At 1 year after cessation, LDL levels were reported by Komiyama et al to decrease significantly, although levels did not change at 3 months [37]. For a long time, it has also been known that smokers have lower HDL levels than nonsmokers. Past studies have shown that HDL levels increase following cessation, and that this increase occurs rapidly, in less than 3 weeks, but with no clear pattern of change thereafter [6]. SC is also reported to be accompanied by a rapid reduction in tobacco smoke carcinogen and toxicant biomarkers. After 3 days of cessation, an >80% reduction has been reported for monohydroxybutyl mercapturic acid, 3-hydroxypropyl mercapturic acid, 4-hydroxybut-2-yl mercapturic acid, S-phenyl mercapturic acid, and 2-hydroxyethyl mercapturic acid. Gradual reduction has been reported for some biomarkers, including a 92% reduction in total NNAL after 42 days [38]. Variable dose-response change in biomarkers of exposure following a reduction in cigarettes smoked per day has also been reported. Some biomarkers, such as plasma nicotine, show a strong dose-response reduction, whereas others, such as plasma thiocyanate, show weaker dose-response reductions [39].

In this study, the list of biological and functional parameters and biomarkers of exposure to be tested was extensive. This list was carefully selected to provide high-quality evidence for the effect of SA on a variety of HPHCs and the health-related effects of smoking that are reversible upon SC. These endpoints will provide further insights into the risk profile of a smoker following abstention from smoking within a short time frame by examining a collection of logical, empirically coherent, and mutually supportive data from multiple clinical risk components across several biological processes, physiological systems, and mechanisms that are known to contribute to the pathogenesis of smoking-related diseases.

The results of this study may offer a valuable point of reference for future assessments of alternative products in the context of tobacco harm reduction, providing scientific evidence of the potential of alternative products to reduce the risk of harm in smokers within a 1-year time frame when compared with continuing to smoke cigarettes. With this aim, cross-study analyses need to be designed carefully to ensure baseline comparability of quitters with the population of smokers switching to the alternative product. Such a comparative approach is valuable for obtaining data on the risk reduction potential of a product alternative to cigarettes earlier than epidemiological studies, which require long-term assessment to provide data.

### Limitations

The design and approach used in the present study should be considered in light of its limitations. One limitation was related to the difficulty of recruiting smokers who were both willing and motivated to quit smoking, completely and continuously, for 1 year. In addition, the uncontrolled before and after study design and the lack of a control arm with no intervention might require careful interpretation of the outcomes of this study. The results of this study should be interpreted with caution owing to the lack of control of potential confounding factors. Factors such as concomitant medications, lifestyle, and weight increase can cause changes in lipid metabolism and other biochemical processes following SC [40].

### Conclusions

This study was designed to provide an extensive dataset on changes in biomarkers of effect and biomarkers of exposure after 12 months of continuous SA. Therefore, it will provide a comprehensive overview of the beneficial short-term health effects that occur over the course of 1 year in a smoker who ceases smoking. The results from this study will complement existing evidence for the benefits of cessation. In the context of smoking harm reduction, these results may be used as a benchmark for the future evaluation of alternative products to cigarettes and to supplement the existing literature on the biological and functional health effects of SC.

## Abbreviations

CVD: cardiovascular disease
FEV1: forced expiratory volume in one second
FVC: forced vital capacity
HPHC: harmful and potentially harmful constituent
NRT: nicotine replacement therapy
PMI: Philip Morris International
PMI R&D: Philip Morris International Research & Development
SA: smoking abstinence
SC: smoking cessation
WBC: white blood cell
WHO: World Health Organization

## References

[1] US Department of Health and Human Services Staff (2010). How Tobacco Smoke Causes Disease: The Biology and Behavioral Basis for Smoking-Attributable Disease: A Report of the Surgeon General.

[2] World Health Organization (2002). The Tobacco Atlas 2002.

[3] Waldum HL, Nilsen OG, Nilsen T, Rørvik H, Syversen V, Sanvik AK, Haugen OA, Torp SH, Brenna E (1996). Long-term effects of inhaled nicotine. Life Sci.

[4] Royal College of Physicians (2007). Royal College of Physicians.

[5] US Department of Health and Human Services Staff (2010). How Tobacco Smoke Causes Disease: The Biology and Behavioral Basis for Smoking-attributable Disease : a Report of the Surgeon General.

[6] Forey BA, Fry JS, Lee PN, Thornton AJ, Coombs KJ (2013). The effect of quitting smoking on HDL-cholesterol - a review based on within-subject changes. Biomark Res.

[7] Pezzuto A, Spoto C, Vincenzi B, Tonini G (2013). Short-term effectiveness of smoking-cessation treatment on respiratory function and CEA level. J Comp Eff Res.

[8] Lee PN (2013). The effect of reducing the number of cigarettes smoked on risk of lung cancer, COPD, cardiovascular disease and FEV(1)--a review. Regul Toxicol Pharmacol.

[9] Wannamethee SG, Lowe GDO, Shaper AG, Rumley A, Lennon L, Whincup PH (2005). Associations between cigarette smoking, pipe/cigar smoking, and smoking cessation, and haemostatic and inflammatory markers for cardiovascular disease. Eur Heart J.

[10] Xu X, Weiss ST, Rijcken B, Schouten JP (1994). Smoking, changes in smoking habits, and rate of decline in FEV1: new insight into gender differences. Eur Respir J.

[11] Saareks V, Ylitalo P, Alanko J, Mucha I, Riutta A (2001). Effects of smoking cessation and nicotine substitution on systemic eicosanoid production in man. Naunyn Schmiedebergs Arch Pharmacol.

[12] Frost-Pineda K, Liang Q, Liu J, Rimmer L, Jin Y, Feng S, Kapur S, Mendes P, Roethig H, Sarkar M (2011). Biomarkers of potential harm among adult smokers and nonsmokers in the total exposure study. Nicotine Tob Res.

[13] Mobarrez F, Antoniewicz L, Bosson JA, Kuhl J, Pisetsky DS, Lundbäck M (2014). The effects of smoking on levels of endothelial progenitor cells and microparticles in the blood of healthy volunteers. PLoS One.

[14] Oguogho A, Lupattelli G, Palumbo B, Sinzinger H (2000). Isoprostanes quickly normalize after quitting cigarette smoking in healthy adults. Vasa.

[15] Rodu B (2011). The scientific foundation for tobacco harm reduction, 2006-2011. Harm Reduct J.

[16] Institute of Medicine (2012). Scientific Standards for Studies on Modified Risk Tobacco Products.

[17] World Medical Association (WMA) (2013). World Medical Association.

[18] (2016). ICH E6 (R2).

[19] Zingg S, Collet T, Locatelli I, Nanchen D, Depairon M, Bovet P, Cornuz J, Rodondi N (2016). Associations between cardiovascular risk factors, inflammation, and progression of carotid atherosclerosis among smokers. Nicotine Tob Res.

[20] Reddy AV, Killampalli LK, Prakash AR, Naag S, Sreenath G, Biraggari SK (2016). Analysis of lipid profile in cancer patients, smokers, and nonsmokers. Dent Res J (Isfahan).

[21] Moracco KE, Morgan JC, Mendel J, Teal R, Noar SM, Ribisl KM, Hall MG, Brewer NT (2016). "My First Thought was Croutons": Perceptions of Cigarettes and Cigarette Smoke Constituents Among Adult Smokers and Nonsmokers. Nicotine Tob Res.

[22] Prochaska JO, DiClemente CC (1983). Stages and processes of self-change of smoking: toward an integrative model of change. J Consult Clin Psychol.

[23] Ashley DL, Burns D, Djordjevic M, Dybing E, Gray N, Hammond SK, Henningfield J, Jarvis M, Reddy KS, Robertson C, Zaatari G, WHO Study Group on Tobacco Product Regulation (2008). The scientific basis of tobacco product regulation. World Health Organ Tech Rep Ser.

[24] (2012). US Food and Drug Administration.

[25] Haziza C, de La Bourdonnaye Guillaume, Skiada D, Ancerewicz J, Baker G, Picavet P, Lüdicke F (2016). Evaluation of the Tobacco Heating System 2.2. Part 8: 5-Day randomized reduced exposure clinical study in Poland. Regul Toxicol Pharmacol.

[26] Fagerström K, Russ C, Yu C, Yunis C, Foulds J (2012). The Fagerström Test for Nicotine Dependence as a predictor of smoking abstinence: a pooled analysis of varenicline clinical trial data. Nicotine Tob Res.

[27] Ferguson J, Bauld L, Chesterman J, Judge K (2005). The English smoking treatment services: one-year outcomes. Addiction.

[28] Scanlon PD, Connett JE, Waller LA, Altose MD, Bailey WC, Buist AS, Tashkin DP, Lung Health Study Research Group (2000). Smoking cessation and lung function in mild-to-moderate chronic obstructive pulmonary disease. The Lung Health Study. Am J Respir Crit Care Med.

[29] SRNT Subcommittee on Biochemical Verification (2002). Biochemical verification of tobacco use and cessation. Nicotine Tob Res.

[30] Berg CJ, Schauer GL, Ahluwalia JS, Benowitz NL (2012). Correlates of NNAL levels among nondaily and daily smokers in the college student population. Curr Biomark Find.

[31] Schuit E, Panagiotou OA, Munafò MR, Bennett DA, Bergen AW, David SP (2017). Pharmacotherapy for smoking cessation: effects by subgroup defined by genetically informed biomarkers. Cochrane Database Syst Rev.

[32] Graham AL, Zhao K, Papandonatos GD, Erar B, Wang X, Amato MS, Cha S, Cohn AM, Pearson JL (2017). A prospective examination of online social network dynamics and smoking cessation. PLoS One.

[33] White MA, Ivezaj V, Grilo CM (2017). Evaluation of a web-based cognitive behavioral smoking cessation treatment for overweight/obese smokers. J Health Psychol.

[34] Xu T, Holzapfel C, Dong X, Bader E, Yu Z, Prehn C, Perstorfer K, Jaremek M, Roemisch-Margl W, Rathmann W, Li Y, Wichmann HE, Wallaschofski H, Ladwig KH, Theis F, Suhre K, Adamski J, Illig T, Peters A, Wang-Sattler R (2013). Effects of smoking and smoking cessation on human serum metabolite profile: results from the KORA cohort study. BMC Med.

[35] Yoon C, Goh E, Park SM, Cho B (2010). Effects of smoking cessation and weight gain on cardiovascular disease risk factors in Asian male population. Atherosclerosis.

[36] King CC, Piper ME, Gepner AD, Fiore MC, Baker TB, Stein JH (2017). Longitudinal impact of smoking and smoking cessation on inflammatory markers of cardiovascular disease risk. Arterioscler Thromb Vasc Biol.

[37] Komiyama M, Shimada S, Wada H, Yamakage H, Satoh-Asahara N, Shimatsu A, Akao M, Morimoto T, Takahashi Y, Hasegawa K (2016). Time-dependent changes of atherosclerotic LDL complexes after smoking cessation. J Atheroscler Thromb.

[38] Carmella SG, Chen M, Han S, Briggs A, Jensen J, Hatsukami DK, Hecht SS (2009). Effects of smoking cessation on eight urinary tobacco carcinogen and toxicant biomarkers. Chem Res Toxicol.

[39] Theophilus EH, Coggins CR, Chen P, Schmidt E, Borgerding MF (2015). Magnitudes of biomarker reductions in response to controlled reductions in cigarettes smoked per day: a one-week clinical confinement study. Regul Toxicol Pharmacol.

[40] Iida M (2016). Weight gain after smoking cessation and atherosclerotic low-density lipoprotein marker. J Atheroscler Thromb.

